# Personal

**Published:** 1889-11

**Authors:** 


### Personal.

---

Dr. Frank S. Billings, late in charge of the patho-biological laboratory of the State University of Nebraska, has removed to Chicago, Ill., to resume his life-work—the mastery of the non-recurrent diseases of children—scarlet fever, mumps, measles, whooping-cough; and he hopes to ultimately establish free hospitals in Chicago for the treatment of these diseases among the poor.

Dr. Billings has fitted up a laboratory at 3600 Michigan avenue, in which he proposes to manufacture virus for the inoculation of swine against hog cholera, and to continue the study of that subject in all its branches. The importance of such a laboratory to the stock breeders of the country cannot well be over-estimated.

---

Dr. W. P. Manton was elected President of the Detroit Gynecological Society at its fifth annual meeting held Wednesday evening, October 23, 1889. The other officers of this flourishing society, chosen for 1890, were: Vice-President, A. W. Turvie, M. D.; Secretary and Treasurer, W. R. Chittick, M. D.

DR. CHARLES P. CLARK, a graduate of the Toronto University, has been appointed Demonstrator in Chemistry in the Niagara University Medical College. Dr. Clark has opened his office at No. 70 West Genesee street.

---

DR. E. T. DORLAND, accompanied by his son, is enjoying the delights of foreign travel. He will return in November, and resume his professional work in this city.

---

DR. JOSEPH C. GREENE has returned from his tour around the world, and resumed his practice at 124 East Swan street, in this city.

